# Supplementary material for: Integrated multifactor assessment of road geometry, vehicle types and weather diversity on bilateral transverse slopes: Bridging gaps in dynamic modeling
Source: PLoS One. 2026 May 5;21(5):e0340887. doi: 10.1371/journal.pone.0340887 (PMC13143120; doi:10.1371/journal.pone.0340887)
Supplement: S1 File — (ZIP) [file pone.0340887.s001.zip › Supporting Information.docx]

Dear Editor

We thank the editor for requesting clarification regarding the Data Availability Statement.

We confirm that all raw data required to replicate the results of this study are included within the manuscript and its Supporting Information files. Specifically, the submission contains the complete minimal data set as defined by PLOS, including:

- The underlying numerical values used to compute all reported sections.
- The complete datasets used to generate all tables and figures presented in the manuscript (e.g., stopping distances, speed profiles, acceleration/deceleration trajectories, and recommended speed limits under different rainfall intensities and longitudinal slopes);
- All model parameters, input assumptions, and simulation settings necessary to reproduce the analyses.

In this section, all input parameters, including route geometry, road gradient, vehicle, driver behavior during lane changes, vehicle speed, and weather conditions, which are related to the modeling performed in this research, are presented.

***As this study is based on a deterministic simulation framework rather than empirical human-subject or proprietary data, no additional raw datasets beyond those provided are required to independently replicate the findings. Accordingly, the existing Data Availability Statement accurately reflects the availability of the minimal data set.***

In this research, the vehicle's performance is examined for its stability against vertical acceleration, yaw angle, horizontal force, vertical force, yaw rate, roll angle, lateral acceleration, slip angle, wheel-road angle, and steering angle under different road geometry, weather, and speed conditions.

The road under study has no longitudinal slope, and the design speeds are 80, 90, 110 and 120 km/h.

Three types of weather are considered for the study. These include dry, rainy and snowy weather, and the equivalent friction coefficient between the wheel and the road for these three types of weather is 0.9, 0.5 and 0.28, respectively.

In this study, the lateral slope is 0%, 1.5%, 2%, and 2.5%, where 0% is for when the vehicle does not change its lateral slope when changing lanes, meaning this slope represents all one-way lateral slopes.

Due to the wide variety of vehicles and the differences in their dimensions, weight, and dynamic characteristics, this study uses three types of vehicles commonly found in articles and references.

| Value | Parameter |
| --- | --- |
| 4220mm | Length |
| 1880mm | Width |
| 1800mm | Height |
| 2950mm | Wheelbase |
| 390mm | Ground Clearance |
| 720mm | Center of Mass Height |
| 1650kg | Weight |

| Value | Parameter |
| --- | --- |
| 4250mm | Length |
| 1880mm | Width |
| 1480mm | Height |
| 3050mm | Wheelbase |
| 320mm | Ground Clearance |
| 530mm | Center of Mass Height |
| 1650kg | Weight |

Supporting information for suv:

Supporting information for truck:

| Value (Cabin) | Value (Cargo Section) | Parameter |
| --- | --- | --- |
| - | 3000mm | Length |
| 2438mm | 2000mm | Width |
| - | 3200mm | Height |
| 2030mm |  | Wheelbase |
| 510mm | 510mm | Ground Clearance |
| 1173mm | 1800mm | Center of Mass Height |
| 4455kg | 6789kg | Weight |

Using the two simulation softwares CarSim and TrackSim, it is possible to simulate at constant or variable speed, with or without changing direction, with or without using brakes. In this research, the type without changing speed, when changing lanes, has been simulated.

In this research, when changing lanes, three types of weather are considered for the road where the lane change occurred, which include dry, rainy and snowy weather. To simulate weather in simulation software, the change in friction between the road and the wheels has been used. The friction coefficients have been applied in such a way that the friction coefficient of 0.9 is considered for a dry road, the friction coefficient of 0.5 for a rainy road, and the friction coefficient of 0.28 for a snowy road.

After defining and entering the required inputs of the scenarios based on the above content in the simulation software, we also define the output graphs required for the research including steering angle, wheel and road angle, slip angle, lateral acceleration, rollover angle, torque rate, vertical forces, horizontal forces, torque angle and vertical acceleration of the vehicle in the software and this is the last modeling step to obtain the output and results. The maximum and minimum values are extracted from each output and finally these values are converted into graphs based on speed, lateral slope or friction coefficient.

I hope the above data is sufficient, but as mentioned in the article, any additional information and data can be provided.

Best regards.
