# Supplementary material for: Integrated multifactor assessment of road geometry, vehicle types and weather diversity on bilateral transverse slopes: Bridging gaps in dynamic modeling
Source: PLoS One. 2026 May 5;21(5):e0340887. doi: 10.1371/journal.pone.0340887 (PMC13143120; doi:10.1371/journal.pone.0340887)
Supplement: Attachment — Submitted filename: Response to Reviewers.docx [file pone.0340887.s003.docx]

**The authors sincerely appreciate the editor and reviewers for their thorough review and insightful comments on our manuscript. The feedback has substantially contributed to strengthening the motivation, methodology, and presentation of our study on lane-changing safety under bilateral transverse slopes. All comments have been carefully considered, and detailed responses along with corresponding changes in the manuscript are provided below on a point‑by‑point basis.**

**Reply to reviewer 1's comments**

Comment 1: The motivation needs to be strengthened. The introduction asserts that roadway transverse slope affects safety, but the manuscript does not clearly explain why this specific geometry problem is urgent or understudied. The authors should contextualize why bilateral slope instability is a priority compared to other geometric factors (e.g., superelevation transitions, crossfall variation, or surface friction). Also, keywords were listed twice in the abstract; pick one set in the revised manuscript.

Thank you for this constructive suggestion. In the revised manuscript, the Introduction has been expanded to clarify why bilateral transverse slopes on suburban roads represent an important yet understudied safety issue compared with other geometric elements such as curve radius, superelevation transitions, and surface friction. We now emphasize that bilateral crossfall is widely used for drainage on nominally straight segments where lane changes are frequent, while existing studies and design criteria are largely based on unidirectional superelevation or curve‑related geometry. The revised text explicitly highlights the lack of integrated analyses combining bilateral slope level, vehicle type, speed, and adverse weather for lane‑changing maneuvers, and explains how our simulation framework is designed to fill this gap (see Introduction, line 48-55 and 141-147).

In addition, we have corrected the abstract by removing the unrelated set of keywords (“accident severity, spatiotemporal analysis, machine learning, metaheuristic algorithm, random forest”) and retaining a single, consistent keyword list focused on the present study (“Bilateral transverse slope; lane change; vehicle dynamics model; road safety; simulation”).

2. Several recent studies that investigate the link between geometry, vehicle dynamic stability, and crash/injury outcomes are missing. The authors should incorporate work in accident severity modeling and spatiotemporal risk analysis to properly anchor the real-world safety relevance of the simulations such as <https://doi.org/10.1080/23249935.2025.2516817>, and <https://doi.org/10.1016/j.aap.2025.108277> .

We appreciate this helpful comment and agree that connecting our simulation study to recent findings on accident severity and spatiotemporal risk enhances the real‑world relevance of the work. In the revised manuscript, we have expanded the Literature Review to discuss recent contributions in accident severity modeling and spatiotemporal safety analysis that explicitly link roadway geometry, vehicle dynamics, and crash/injury outcomes, including the suggested studies. These works are now cited to emphasize that cross‑sectional design and stability‑related mechanisms are important determinants of crash severity and spatial–temporal risk patterns. We also clarify how our simulation framework complements this literature by providing detailed dynamic responses for different vehicle types and weather conditions on bilateral transverse slopes used in suburban cross‑sections (see Literature Review, lines 240–248, and Introduction, lines 128-130).

3. The manuscript states that vehicle behavior 'changes with slope,' but does not specify which outcomes matter most for safety and why. The authors should explicitly identify which performance measures (e.g., yaw stability, rollover risk, lateral load transfer) drive crash likelihood.

Thank you for this important comment. In the revised manuscript, we now explicitly identify and justify the key performance measures that are used as safety indicators. Specifically, we highlight yaw stability (yaw rate and yaw moment), rollover risk (roll angle as a proxy for lateral load transfer and incipient rollover), and lateral stability (slip angle and lateral acceleration), with steering angle and torsional moment treated as complementary measures of control effort and rotational behavior. These variables are now described in the Introduction and Methodology as direct precursors to loss‑of‑control, rollover, and sideslip‑related crashes, particularly at higher speeds and under low‑friction conditions. Furthermore, in the Results, statements about vehicle behavior “changing with slope” have been revised to explicitly reference the affected indicators (e.g., roll angle, slip angle, yaw moment) and to interpret their implications for crash likelihood (see Introduction, lines 141-147; Methodology – Data Analysis, lines 385- 390; and conclusion lines 666-680).

4. Values selected in CarSim/TruckSim (vehicle mass, center of gravity, lane-change steering profile, speed input) are presented without referencing empirical data or prior experimental standards. The study should justify these values or provide sensitivity analysis to demonstrate robustness.

Thank you for this valuable suggestion. In the revised manuscript, we have added a dedicated paragraph in the Methodology section ("Simulation Setup") justifying all key input parameters with references to CarSim/TruckSim's validated standard vehicle databases (derived from manufacturer data and real-world testing) and standardized maneuvers (e.g., ISO 3888-2 double lane change). Speeds were selected to reflect suburban highway conditions, and a sensitivity analysis for center of gravity (±10%) and friction (±0.1) confirms the robustness of primary findings such as slope-induced increases in rollover angle and yaw moment (see Methodology, new paragraph under " Vehicle Selection"). These enhancements demonstrate that the simulation outputs are reliable and representative of physical vehicle behavior

5. The paper presents only simulated results, and no comparison is made to observed crash patterns or empirical instability thresholds. Without any validation or triangulation, it is impossible to assess whether the findings have practical road design implications.

We appreciate this important comment and acknowledge the limitation of presenting simulation results without direct empirical crash data comparison. To address this, we have added clarifying text in the Discussion section to highlight that CarSim and TruckSim models used here are extensively validated against real-vehicle tests and standardized maneuvers, ensuring reliable dynamic behavior representation. Additionally, we note that the key performance measures and thresholds observed align with values documented in prior field and experimental studies relating to crash risk and instability. We also explicitly state that future work will incorporate empirical crash and severity data to triangulate these simulation findings, thereby enhancing their applicability to road design and safety practices (see Line 727-735). This response contextualizes the simulations within existing validation frameworks and acknowledges ongoing steps towards broader empirical integration.(future work lines 759-763).

6. The results section lists trends (e.g., higher slope → increased rollover angle), but does not translate them into meaningful conclusions for roadway engineers and policy makers. The authors should explain how much slope change is significant enough to alter safety outcomes.

Thank you for highlighting this key point. In the revised Results and conclusion sections, we now translate simulation trends into actionable thresholds for engineers: rollover angles and lateral accelerations increase significantly (>15-20%) at slopes ≥2.5% for trucks/SUVs above 90 km/h on low-friction surfaces, approaching empirical rollover thresholds (0.8-1.2g lateral acceleration) that elevate crash risk. Slopes ≤1.5% show negligible effects (<5% change in stability metrics) and align with safe design practice, while 2-2.5% warrants caution in mixed-traffic suburban applications. These quantitative guidelines are now presented for direct use in road design standards and ESC tuning (see CONCLUSION lines 736-748 (

7. The manuscript claims that the results 'provide guidance for road design,' but no actionable thresholds, design limits, or policy recommendations are actually provided. The conclusion should be revised to reflect the exploratory nature of the study or include explicit guidance.

Thank you for this important observation. In response, the Conclusion section has been revised to explicitly acknowledge the exploratory and simulation-based nature of the current study while simultaneously providing initial actionable thresholds and design recommendations rooted in the simulation results. Specifically, we highlight the critical 2.5% bilateral transverse slope limit for heavy vehicles traveling above 90 km/h in adverse conditions and encourage the incorporation of advanced vehicle stability systems like ESC. We also outline the need for future empirical validation to strengthen these guidance points for practical infrastructure design and policy. This revision clarifies the scope of the study and the preliminary nature of design guidance, enhancing transparency and usefulness for practitioners (see Conclusion, 727-735).

**Reply to referee 2's comments**

1. The simulated scenarios do not include curved roads or mixed traffic flows, which deviates from real-world road environments. It is recommended to supplement simulations of curved transverse slope scenarios to enhance the practicality of the research.

Thank you for this insightful comment regarding scope limitations. This point was already explicitly addressed in the revised manuscript's new **"** **Future Research and limitation" subsection**.

2. The assumptions about driver behavior are overly simplified, failing to account for real-world operations such as emergency braking and steering deviations. Variables for complex driving behaviors should be added to improve model authenticity.

Thank you for this valuable suggestion regarding driver behavior modeling. In the revised manuscript, we have clarified in the Methodology section ("Driver Behavior" and new paragraph under it) that the study employs the standardized ISO 3888-2 double lane change protocol with constant speed and no braking, which is the established industry standard for isolating geometric effects on vehicle stability in CarSim/TruckSim validation studies (R² > 0.97 correlation with physical tests). This controlled input design enables clear attribution of stability changes to bilateral transverse slopes rather than confounding driver inputs.To address realism concerns, we have added a new "Limitations and Future Work" subsection in the Discussion.

3. The results section lacks significance analysis of parameter changes. It is suggested to supplement Analysis of Variance (ANOVA) or correlation analysis to clarify the statistical significance of the impacts of slope, speed, weather, and other factors on stability parameters.

Thank you for this constructive recommendation. In response, we have incorporated a statistical analysis subsection within the Results section (new paragraph and Table 4) performing Analysis of Variance (ANOVA) to quantitatively assess the significance of independent factors—bilateral transverse slope, speed, weather condition, and vehicle type—on key stability parameters including rollover angle, slip angle, yaw moment, and steering angle. Results indicate that slope and speed interactions significantly (p < 0.01) influence rollover and yaw dynamics, while weather notably affects slip angles and lateral acceleration. The findings quantitatively substantiate our qualitative trend observations and clarify the relative effect sizes of these variables, enhancing the rigor and interpretability of the conclusions.

4. Quantitative criteria for rollover thresholds of different vehicle types (especially trucks) are not clearly defined.

## Thank you for raising this important point regarding rollover threshold criteria. In the revised manuscript, we have incorporated quantitative rollover thresholds grounded in empirical and regulatory research. Passenger cars and SUVs generally exhibit lateral acceleration rollover thresholds between 0.8 and 1.2g, reflecting their lower center of gravity and chassis design. In contrast, heavy trucks demonstrate significantly lower thresholds, typically around 0.25 to 0.35g, depending on loading and suspension characteristics, due to their higher center of gravity and mass distribution. These thresholds provide critical benchmarks for interpreting simulated rollover angles and lateral accelerations in our study. Specifically, rollover angles corresponding to lateral accelerations near or exceeding 0.3g for trucks are considered indicative of elevated rollover risk. This quantitative contextualization enhances the practical relevance of our results by aligning simulated stability metrics with established safety limits across vehicle classes (see Effect of Lateral Slope Changes on Rollover Angle section lines 477-486).

5. The analysis of weather factors only focuses on friction coefficients, ignoring key variables such as visibility.

Thank you for this valuable observation. In the revised manuscript, we acknowledge this limitation explicitly in the newly added Limitations and Future Work subsection. While the current study models weather effects primarily through surface friction coefficients corresponding to dry, rainy, and snowy conditions—which critically influence vehicle dynamic stability—other weather-induced factors such as reduced visibility, driver perception delays, and increased reaction times are outside the current simulation scope. We recognize that these variables can significantly affect real-world lane-changing behavior and crash risk. Future research directions will extend the simulation framework to incorporate driver visibility impairments and sensor-based responses, potentially through coupling with traffic simulation or driver behavior models to capture comprehensive environmental impacts on safety. This enhancement will improve the ecological validity of the findings for adverse weather conditions beyond frictional characteristics alone.

6. Recommendations for ESC optimization for trucks are overly vague. Specific parameter adjustment plans (e.g., ESC intervention thresholds under different slopes) should be provided based on simulation data to enhance engineering operability.

Thank you for this practical suggestion. In the revised manuscript, we have added a new "ESC Optimization Guidelines" in the conclusion in lines 697-704 providing specific, simulation-derived ESC intervention thresholds for trucks:

ESC Intervention Thresholds for Trucks on Bilateral Transverse Slopes:

Slopes ≤1.5%: Standard ESC thresholds (lateral accel. ≥0.4g, yaw rate ≥8°/s) sufficien

Slopes 2.0–2.5%: Lower intervention thresholds recommended (lateral accel. ≥0.35g, yaw rate ≥6°/s) due to 15–20% rollover angle increase observed at 90+ km/h

Snowy conditions (μ=0.28): Activate ESC at lateral accel. ≥0.30g (truck rollover threshold) and yaw rate ≥5°/s to counter amplified slip angles.

These thresholds, derived from simulation data showing critical stability margins at 0.3–0.35g lateral acceleration for trucks, enable OEMs to recalibrate ESC algorithms for slope-compensated intervention timing, enhancing rollover prevention without excessive activation on normal roads.

We appreciate the thorough and constructive feedback from the reviewers, which has significantly improved the clarity, rigor, and practical relevance of our study; we trust that the detailed responses and manuscript revisions fully address their concerns and enhance the contribution of our work to the field of road safety engineering.
